# ‘We just dilute sugar and give’ health workers’ reports of management of paediatric hypoglycaemia in a referral hospital in Malawi

**DOI:** 10.1080/16549716.2018.1491670

**Published:** 2018-07-17

**Authors:** Cecilia Lindsjö, Chawanangwa Mahebere Chirambo, Josephine Langton, Queen Dube, Tim Baker, Helena Hildenwall

**Affiliations:** a Global Health – Health System and Policy Research Group, Department of Public Health Sciences, Karolinska Institutet, Stockholm, Sweden; b Department of Paediatrics, College of Medicine, University of Malawi, Blantyre, Malawi; c Astrid Lindgren Children’s Hospital, Karolinska University Hospital, Stockholm, Sweden

**Keywords:** hypoglycemia, children, critical illness, Africa, health workers

## Abstract

**Background**: Acutely sick children in resource-constrained settings who present with hypoglycaemia have poor outcomes. Studies have questioned the current hypoglycaemia treatment cut-off level of 2.5 mmol/l. Improved knowledge about health workers’ attitudes towards and management of hypoglycaemia is needed to understand the potential effects of a raised cut-off level.

**Objective**: This research explored health workers’ perceptions about managing acutely ill children with hypoglycaemia in a Malawian referral hospital. A secondary objective was to explore health workers’ opinions about a potential increase in the hypoglycaemia cut-off level.

**Methods**: We used a qualitative design with semi-structured individual interviews performed with health workers in the Paediatric Accident and Emergency Unit at Queen Elizabeth Central Hospital, Malawi, in October 2016. Data were analysed using latent content analysis. Ethical approval was obtained from the University of Malawi, College of Medicine Research and Ethics Committee P.01/16/1852.

**Results**: Four themes were formed that described the responses. The first, ‘Critical and difficult cases need easy treatment’, showed that health workers perceived hypoglycaemia as a severe condition that was easily manageable. The second, ‘Health system issues’, revealed challenges relating to staffing and resource availability. The third, ‘From parental reluctance to demand’, described a change in parents’ attitudes regarding intravenous treatments. The fourth, ‘Positive about the change but need more information’, exposed health workers’ concerns about potential risks of a raised cut-off level for hypoglycaemia treatment, as well as benefits for the patients.

**Conclusions**: Health workers perceived hypoglycaemia as a severe condition that is easy to manage when the required equipment and supplies are available. Due to the common lack of test equipment and dextrose supplies, health workers have adopted alternative strategies to diagnose and manage hypoglycaemia. A change to the hypoglycaemia treatment cut-off level raised concerns about potential risks, but was also thought to be of benefit for some patients.

## Background

Hypoglycaemia in acutely sick children is associated with poor clinical outcomes [1] and potentially long-term negative effects on neurodevelopment [2]. The World Health Organization (WHO) defines paediatric hypoglycaemia outside the neonatal period as a blood glucose value of <2.5 mmol/l or <3 mmol/l in severely malnourished children [3]. However, recent studies have found increased mortality also in children with low glycaemia, defined as an admission glucose level between 2.5 and (4.4–)5.0 mmol/l [1,4], and this has led to a questioning of the current cut-off level for hypoglycaemia treatment [5,6]. A randomised clinical trial to assess the mortality impact of an increased blood glucose treatment cut-off level, from 2.5 mmol/l to 5.0 mmol/l in children 1 month to 5 years, is ongoing [7].

For a changed cut-off level to have an impact on outcomes, health workers need to both trust management guidelines and have the ability to adhere to recommendations. Symptoms of hypoglycaemia are non-specific, and hypoglycaemia diagnosis and management may be challenging where critical care is limited [8]. Little is known about how health workers experience the management of hypoglycaemic children in resource-constrained settings. We aimed to explore health workers’ perceptions about managing acutely ill children with hypoglycaemia and their opinions about raising the treatment cut-off level.

## Methods

### Setting

Queen Elizabeth Central Hospital (QECH) is a referral hospital in Blantyre, Malawi. The hospital serves a predominately rural population and manages 26,000 paediatric admissions annually. The paediatric Accident and Emergency (A&E) department has a well-defined triage system whereby children with WHO emergency signs [9] are sent to a resuscitation room. Nurses and clinical officers are present in the A&E department 24 hours a day and intern doctors are usually available to assess patients. Paediatric registrars (specialist trainee doctors) staff the paediatric resuscitation room during office hours.

QECH uses a locally adapted WHO pocket book of hospital care for children [9], as well as locally developed guidelines for the management of severely sick children. The WHO Emergency Triage, Assessment and Treatment (ETAT) guidelines [3] for the structured management of acutely sick children were developed at QECH and have been in use since 2001. Paediatric in-hospital mortality is estimated at 5% (personal communication, Head of Department of Paediatrics at QECH) and the Malawi under-five mortality rate has declined from 149.2 per 1000 live births in 2002 to 59.1 per 1000 live births in 2015 [10].

### Design

This qualitative research was conducted as a formative study within the framework of the SugarFACT trial that is currently ongoing at QECH [7].

### Study population

All staff (nurses and clinicians) who were rotating through the paediatric resuscitation room at the time of the study were approached face to face and invited to participate in interviews during working hours. All 12 health workers consented to participate in the interviews.

### Data collection

Semi-structured individual interviews were completed with health workers until saturation was reached, prior to the sensitisation and start of the trial in October 2016. The interview guide was designed by the research team and three pilot interviews were completed. The pilot interviews were included in the analysis as the questions remained the same and only their order was changed for a better flow through the interviews. The health workers were asked open-ended questions regarding their experience of hypoglycaemia, which patients they would consider or reject for treatment, preferred treatment, and expected outcomes in order to give interviewees an opportunity to explain their experiences with their own words (see Annex 1). The interviews took between 19 and 35 minutes and were conducted in a private room in the hospital. A Malawian social scientist (CMC) who speaks fluent English and Chichewa, the local language, and has an understanding of the local context, performed the interviews. Interviews were mainly conducted in English, though Chichewa was encouraged if needed to clarify discussion points.

### Data management and analysis

Interviews were audio recorded and transcribed by the same person who completed the interviews (CMC). All transcripts were carefully read multiple times by the lead author (CL) and were analysed using content analysis as described by Graneheim and Lundman [11]. Meaning units relevant to the aim of the study were identified to inform an initial coding scheme. Preliminary codes were further refined and applied back to transcripts. These codes were discussed and revised by two authors (CL, HH) and were then grouped into mutually agreed categories to describe response patterns. These categories were further refined into a set of final themes. A Microsoft Excel worksheet was used for data management.

### Ethical considerations

The participants were informed about the study and that their participation would be voluntary, and were given the opportunity to ask questions before they were asked for consent. Ethical approval was obtained from the University of Malawi, College of Medicine Research and Ethics Committee P.01/16/1852.

## Results

A total of nine women and three men were interviewed. The participants’ age and work experience ranged from 20 to above 50 years, and from 6 months to >30 years, respectively.

Four themes were formed that describe the response patterns: (1) critical and difficult cases need easy treatment, (2) health system issues, (3) from parental reluctance to demand, and (4) positive for the change but need more information.

### Critical and difficult cases need easy treatment

#### Identifying patients with hypoglycaemia

Health workers reported that signs that would make them suspect hypoglycaemia in children were coma, convulsions, not responding, reduced conscious level, floppiness, lethargy, bad shape, weakness, severe dehydration, marasmus or kwashiorkor, and restlessness.

The leading manifestation anyway would be convulsion, they might come in seizing but also sometimes they will just come lethargic so basically they won’t respond when you call their name but they might respond when you inflict pain on them [….] (ID4)

The airway–breathing–circulation (ABC) concept was used to assess and identify children with hypoglycaemia, including a modified version of that found in the ETAT guidelines: airway-breathing-circulation-coma-convulsion-dehydration (ABCCCD(*EFG*)) [3], where D(*EFG)* stands for Don’t Ever Forget Glucose. Information from guardians regarding the patient’s nutritional intake during recent days helped to strengthen the suspicion of hypoglycaemia.

*Ok. We ask history from the mother, what’s the problem with the child? She might tell you that the child is just weak. If you are told that the child is just weak then you ask when the child last ate. [….] So when we have that history, it gives you an impression that I think I should first check […] the level of blood sugar. (ID6)*

Blood glucose was reportedly tested in all patients presenting with symptoms suggestive of hypoglycaemia, as well as all patients triaged to the hospital’s resuscitation room. However, at times the diagnostic tools were lacking and hypoglycaemia could not be confirmed. In cases when blood glucose testing was not possible, the recent nutritional history and the presentation of the child would guide decisions on whether to regard the patient as hypoglycaemic.

*Under the Integrated Management of Childhood Illnesses algorithm […] if you are in a situation that you have not tested sugars [and] the child is coming to you with signs of hypoglycaemia, it is safe to treat hypoglycaemia blindly. I have done that before. Babies coming in convulsing, yes I am going to give you diazepam but I would still give you glucose in case you got hypoglycaemia. (ID4)*

#### Treatment decisions

Treatment would be given when a test result confirmed hypoglycaemia. If possible, a clinician would be consulted to decide on the treatment.

*So when it is low we need to give them bolus if it’s severely low those are the ones we give bolus but some we put them on maintenance. For those they can do without we encourage the mother to breastfeed if they are breastfeeding. The ones that are a little bit older we basically give them sugary fluids so basically that’s what we do in managing them. (ID3)*

Treatment was sometimes also considered despite a normal blood glucose level if there was a history of poor feeding during the illness, or if the child was perceived to be very sick – for example in patients with malaria, weakness, or restlessness, or to prevent hypoglycaemia. Some said that a dextrose infusion should not be given if the blood glucose was normal, while others said that a dextrose bolus should be avoided but a maintenance dextrose infusion was acceptable. Intravenous dextrose was the treatment of preference, but if dextrose fluids were out of stock oral administration of Oral Rehydration Solution or sugar diluted in water was suggested. Sublingual administration was not mentioned as a treatment method.

The treatment result was described as a rapid improvement shortly after a bolus had been given. Hypoglycaemia was described, by some of the respondents, as a condition that was easy to treat and as associated with a low mortality rate.

*One thing I like about hypoglycaemia is [the] mortality rate is very low. So I have never had a patient who has died under my watch solely because of hypoglycaemia. (ID4)*

On the other hand, simultaneous hypoglycaemia and shock was said to be difficult to manage. Furthermore, treatment was regarded as crucial and suspected hypoglycaemia was given high priority.

*Babies with hypoglycaemia can die if not helped*. *(ID2)*

### Health system issues

#### Consequences of staff shortages

Interviewees reported that staff shortages were common, and this was perceived to put high demands on nurses to independently manage patients when doctors were busy with other patients. Staff shortages had a negative impact on the ability to monitor intravenous treatments, and therefore Oral Rehydration Solution was sometimes preferred over intravenous fluids. Another issue was the high staff turnover, which caused colleagues not to trust each other’s ability to dilute dextrose into the desired concentration.

*when somebody says: "I have dissolved  [dextrose]", I just don’t know [if I can trust them]. I have trust issues when it comes to medical practice [laughter]. I have to make sure that they [colleagues] have done it [diluted dextrose]. It is not everyone who knows how to do these things. We change nurses for most of the times. Interns change. Some doctors are junior doctors and are not aware of what is happening, it takes time. Even with me it took time to master it. (ID4)*

#### Availability of supplies

Some health workers reported a reliable availability of dextrose supplies, and the paediatric department seemed to be prioritized within the hospital. However, the majority perceived a lack of specific concentrations of dextrose. The different concentrations placed demands on staff to know how to dilute the required concentration from the currently available strength. In addition, dextrose could be completely out of stock in A&E, while still available at other wards in the hospital.

*we may not have 50% glucose sometimes we may have 25% glucose. Sometimes we don’t have both. Like we have to start searching in other wards. Maybe the pharmacy by then […] do not have [it] so we rush around. By then we are delaying the treatment to the child. So yeah! We have those problems. (ID3)*

Availability of test equipment was perceived to be limited, and the problem included lack of batteries, broken glucometers, and the fact that the pharmacy provided only one kind of test sticks that were not always compatible with the available glucometer.

*but gluco-sticks, working glucometers, sometimes even the cannulas, that is always an issue. (ID4)*

#### Competence limitations

Health workers sometimes expressed insecurity regarding conducting the clinical assessment to identify patients with hypoglycaemia in the absence of test equipment.

*I can say sometimes you cannot diagnose as hypoglycaemia unless you check the sugars, sometimes it looks like severe dehydration because the child is in a very lethargic condition, very weak with severe dehydration. (ID11)*

Awareness of different treatment levels for hypoglycaemia in normal and malnourished children was limited. It took time to master dissolving the right dextrose concentration, and it was not easy to perform the calculations – especially in emergency situations. This was therefore perceived to be something health workers had to know off by heart. Guidelines to facilitate the work were seldom mentioned.

Differences between staff in emergency management were acknowledged, and management guidelines were requested.

*we may have differences among ourselves. One would say start with shock another would say start with hypoglycaemia. I remember we have had such discussion. So a proper management guideline [would] be of help. (ID3)*

Feelings of insecurity amongst colleagues were reported in situations when patients were assessed. Inaccuracies in management were acknowledged when blood glucose testing was sometimes forgotten.

*[If we] see that a child is in a coma or a child is convulsing I normally send the child to resuscitation but still I write ‘please check sugar’. Sometimes the child could go there and members of staff could forget [to test] the sugar. And they would do malaria parasite smears, putting cannulas, putting oxygen forgetting about the sugar. (ID12)*

Health workers reported that most members of staff knew when to give glucose but not how to correctly mix dextrose to the right concentration. Feelings of incompetence, both in oneself and in colleagues, created a feeling of fear of giving the wrong treatment.

### From parental reluctance to demand

The majority of health workers reported no problems regarding parental acceptance of intravenous treatments, and some argued that parents accept the intravenous treatment since they are desperate to get help for their children. Only two health workers stated that parents could express reluctance regarding the insertion of peripheral venous catheters. When faced with parental reluctance in terms of treatment, health workers made efforts to persuade parents that intravenous treatment was indicated. There had reportedly been a recent change in attitudes towards intravenous treatments, with increased acceptance from parents.

*I used to have those problems maybe 4 to 5 years before but now I think […] People are being sensitised and are able to understand why we do that. [….] So right now we don’t see most mothers or most parents denying treatment based on cannulas.* (*ID3)*

Parents requested and had a belief in the superiority of intravenous treatment. This was seen as a potential obstacle in the cooperation with parents, as they could opt out from using Oral Rehydration Solution due to their preference for intravenous treatment.

*I don’t know whether to say fortunately or unfortunately we have a perception I don’t know as Malawians and also elsewhere. Given intravenously that’s when you have had the treatment. (ID4)*

### Positive for the change but need more information

No health worker reported that they had questioned the hypoglycaemia cut-off level, though they sometimes provided hypoglycaemia treatment for patients with a blood glucose above 2.5 mmol/l. Health workers perceived that raising the cut-off blood glucose level for hypoglycaemia treatment would have a potentially negative influence on their work situation. A raise would increase the number of patients in need of treatment and respondents expressed concerns regarding staff shortages.

*At 45 mg/dL* [2.5 mmol/l] *we already have a (staff) shortage. What about at 90* [5 mmol/l], *which is an increase of 100%, so it will be a shortage of the shortage. (ID8)*

However, there were different opinions on whether the change in cut-off would increase the workload, and some of those who claimed it would were still positive about the change.

*No as long as we are helping the patients and they are [doing] fine there is no problem. (ID9)*

Concerns were expressed about potential risks of a raised cut-off for treating hypoglycaemia. It was said that too much dextrose might have a negative impact on the brain cells, cause fluid overload or cause the veins to be too full for the heart to work properly. Some health workers expressed uncertainty on what the potential risks could be.

*I am not sure how biologically it is going to have an impact on the patient. Little children if we give them too much sugar [it] might have an impact on their brain cells [I] am not really sure I [would] need to go back and read […] to comment on that. But then I am worried. If we increase it I am worried. (ID4)*

The change was welcomed, but more information and supplies were requested to manage the change. Positive impacts were also highlighted, and some of the respondents believed that with a raised hypoglycaemia treatment cut-off level children with low glycaemia who are weak and cannot eat will be better treated, and children with hypoglycaemia will not be missed.

## Discussion

To our knowledge, this is the first study to qualitatively explore health workers’ perceptions of hypoglycaemia management in acutely ill children in a resource-constrained setting.

Three main findings emerged from our analysis: (1) hypoglycaemia was perceived to be a severe but easily treatable condition, (2) health workers faced difficulties in diagnosing and managing hypoglycaemia due to staff shortages and limited availability of equipment, and (3) A potential increase in the hypoglycaemia treatment cut-off level raised concerns about increased demands on already overworked staff, but was also thought to be of potential benefit for some patients.

Hypoglycaemia was perceived to be a severe condition: health-workers described severe symptoms in children presenting with hypoglycaemia. Despite this, health workers reported hypoglycaemia to be an easily treatable condition with rapid and visible recovery. Studies from sub-Saharan Africa and other low-income settings have demonstrated a case fatality rate of as much as 41.9% in children with hypoglycaemia on admission [1,4,12], and it has been found to be a predictor of poor outcomes in malaria [13]. It is thus interesting that interviewees reported it as easy to treat. It could be that hypoglycaemia is seen as only part of a general picture and that death is attributed to the overall condition of the child, rather than specifically due to the hypoglycaemia. In resource-constrained countries with weak health systems, care seeking is often delayed due to financial and physical barriers [14,15] that may result in children presenting in late stages of illness. This may be one of the several explanations [12,16–18] why low and hypoglycaemia are common in children in sub-Saharan African hospitals [1,12]. The potential long-term neurological outcomes following hypoglycaemia in acute paediatric illness as described in neonates [19,20] have not been investigated to date.

Difficulties in the diagnosis and management of hypoglycaemia were reported to be due to staff shortages and limited availability of equipment. This needs to be considered in light of the fact that QECH is a referral hospital and may have better staffing and resources than other hospitals in Malawi and neighbouring countries [21,22]. While blood glucose testing is recommended to confirm hypoglycaemia in children with WHO emergency signs according to the ETAT guidelines [3], a lack of test equipment was reportedly common in our study, and probably even more so in lower-level facilities. Health workers used history taking regarding recent nutritional intake to strengthen their suspicion of hypoglycaemia, and this strategy is backed by evidence linking pre-admission fasting with hypoglycaemia [12]. Health workers also discussed the need to prevent hypoglycaemia in fragile patients with normal blood glucose and, in line with ETAT recommendations, would treat some patients blindly when testing was not possible.

Dextrose supplies were reportedly not reliably accessible or were only available in strengths that necessitated mixing to make the desired concentration. The ability to identify hypoglycaemia is not sufficient if the appropriate therapy remains unavailable [23], and this may be a more pronounced problem in lower-level facilities that lack intravenous infusions. When there are complete stock-outs of dextrose infusions, health workers reported that they dissolve sugar in water and give it to the child orally. Sublingual administration was not mentioned, though absorption is faster than via the oral route [24], and the use of sublingual 40% dextrose gel rubbed into the oral mucosa has been demonstrated to be comparable to intravenous dextrose in neonatal hypoglycaemia in a high-income setting [25]. The role of sublingual glucose in acutely sick children with confirmed hypoglycaemia showed promising results in a pilot randomized controlled trial [26]. Though the sample was too small to provide statistically significant results, sublingual sugar may provide an opportunity to treat or avoid hypoglycaemia in resource-constrained settings and the recommended alternative treatment in Integrated Management of Childhood Illness (IMCI) chart booklet should be adapted [24,27].

The suggested change to the cut-off level for hypoglycaemia treatment both raised concerns for patient safety and was thought to be of potential benefit for some patients. Some interviewees referred to risks related to hyperglycaemia, and there is some evidence that admission hyperglycaemia in critically ill children is associated with increased mortality [28–30]. While tight glycaemic control has been practiced in intensive care units with the assumption that hyperglycaemia in the critically ill worsens outcomes, recent studies have shown no difference in mortality or morbidity between severely sick children assigned to a higher or lower target group [31–33]. Although there is little evidence that dangerous hyperglycaemia or poor outcomes result from treating hypoglycaemia, hyperglycaemia, as well as recurrent hypoglycaemia, may be common after dextrose infusions [34]. Considering the imperfect performance of point-of-care glucose testing, an upward adjustment of the treatment threshold could be merited due to the risk of instrument bias [35].

Another concern about an increased treatment cut-off level was the increased demands on staff. This is an important consideration as working in an understaffed facility may cause more staff to leave their current posts [21], which in turn should be considered when contemplating interventions that could add to staff workload.

### Methodological considerations

This study was performed in a large referral hospital, so the transfer of our findings to smaller hospitals that may have a more severe lack of resources and staff should be done with caution. The small sample size and the fact that only one institution was included in the study limits the generalizability of the results. Future studies should explore perceptions and management in smaller health facilities, including health centres.

The majority of interviewees were nurses, who may rely on clinicians for any management decisions, especially in more complicated cases. Still, due to the widespread staff shortages, nurses often manage acutely sick patients by themselves and are the first to initiate treatment. Credibility was strived for via triangulation of the analysis between researchers, and confirmation of validity of findings from staff with experience of working in A&E. All interviews were performed by one person, which minimized differences between the follow-up questions.

### Conclusions

We conclude that health workers perceived hypoglycaemia to be a severe condition that is easy to manage when the required equipment and supplies are available. Health workers had adapted alternative strategies to diagnose and manage hypoglycaemia when test equipment and dextrose supplies were limited. Sublingual administration had not been adopted despite it having been shown to be superior to oral treatment. The suggested change in the treatment cut-off level raised concerns about potential risks and increased demands on already overworked staff, but was also expected to benefit some patients. Future studies should explore perceptions in smaller and less well-equipped health facilities.

## Paper context

Little is known about health workers’ perceptions of managing patients with hypoglycaemia in resource-constrained settings. This study contributes via accounts that shed light on the challenges of delivering care to these patients, and health workers’ solutions to the obstacles they face. Hypoglycaemia was perceived to be a severe but easily treatable condition, though challenges in management related to lack of test equipment and/or treatment were noted. Future studies should explore perceptions in smaller and less well-equipped health facilities.
